# Second exposure to acetaminophen overdose is associated with liver fibrosis in mice

**Published:** 2019-02-06

**Authors:** Mohammad AlWahsh, Amnah Othman, Lama Hamadneh, Ahmad Telfah, Jörg Lambert, Suhair Hikmat, Amin Alassi, Fatma El Zahraa Mohamed, Roland Hergenröder, Tariq Al-Qirim, Steven Dooley, Seddik Hammad

**Affiliations:** 1 Department of Pharmacy, Faculty of Pharmacy, Al-Zaytoonah University of Jordan, P.O. Box 130, Amman 11733, Jordan; 2 Leibniz-Institut für Analytische Wissenschaften - ISAS - e.V., Bunsen-Kirchhoff-Straße 11, 44139 Dortmund, Germany; 3 Molecular Hepatology Section, Department of Medicine II, Medical Faculty Mannheim, Heidelberg University, 68167-Mannheim, Germany; 4 Department of Pathology, Faculty of Medicine, Minia University, 11432-Minia, Egypt; 5 Department of Forensic Medicine and Veterinary Toxicology, Faculty of Veterinary Medicine, South Valley University, 83523-Qena, Egypt

**Keywords:** APAP, metabolic profiling, adaptation

## Abstract

Repeated administration of hepatotoxicants is usually accompanied by liver fibrosis. However, the difference in response as a result of repeated exposures of acetaminophen (APAP) compared to a single dose is not well-studied. Therefore, in the current study, the liver response after a second dose of APAP was investigated. Adult fasted Balb/C mice were exposed to two toxic doses of 300 mg/kg APAP, which were administered 72 h apart from each other. Subsequently, blood and liver from the treated mice were collected 24 h and 72 h after both APAP administrations. Liver transaminase, i.e. alanine amino transferase (ALT) and aspartate amino transferase (AST) levels revealed that the fulminant liver damage was reduced after the second APAP administration compared to that observed at the same time point after the first treatment. These results correlated with the necrotic areas as indicated by histological analyses. Surprisingly, Picro Sirius Red (PSR) staining showed that the accumulation of extracellular matrix after the second dose coincides with the upregulation of some fibrogenic signatures, e.g., alpha smooth muscle actin. Non-targeted liver tissue metabolic profiling indicates that most alterations occur 24 h after the first dose of APAP. However, the levels of most metabolites recover to basal values over time. This organ adaptation process is also confirmed by the upregulation of antioxidative systems like e.g. superoxide dismutase and catalase. From the results, it can be concluded that there is a different response of the liver to APAP toxic doses, if the liver has already been exposed to APAP. A necroinflammatory process followed by a liver regeneration was observed after the first APAP exposure. However, fibrogenesis through the accumulation of extracellular matrix is observed after a second challenge. Therefore, further studies are required to mechanistically understand the so called “liver memory”.

## Introduction

According to the National Center for Health Statistics, liver disease is considered one of the main causes of death worldwide (Pagidipati and Gaziano, 2013[26]). Drug-induced liver injury (DILI) remains the most prevalent cause of liver diseases in the United States (Bernal and Wendon, 2013[4]; Lee, 2017[22]; Ostapowicz et al., 2002[25]). In addition, DILI is thus far considered an unpredictable event that prevents potentially useful therapeutic compounds from entering the market, or results in their early withdrawal (Atienzar et al,. 2016[2]; Godoy et al., 2013[10]). Acetaminophen (N-acetyl-p-aminophenol [APAP]) is the most widely used analgesic and antipyretic, and considered safe within therapeutic doses (Larsen and Wendon, 2014[21]). On the other hand, an APAP overdose leads to severe liver intoxication and hepatocellular necrosis (Ramachandran and Jaeschke, 2017[29]). Unfortunately, APAP remains the most common cause of acute liver failure in Europe, UK and North America, and represents a significant healthcare burden (Craig et al., 2012[7]; Lee, 2017[22]). 

One of the most frequently used experimental models of DILI is using APAP as a model compound. High doses of APAP (e.g. 300 mg/kg in mice) result in the death of a large fraction of hepatocytes, which amount to approximately 40-50 % of the liver mass (Gunawan et al., 2006[13]). This pericentral damage regenerates within 7 days in a well-orchestrated, highly reproducible process. APAP is metabolically bioactivated by cytochrome P450 2E1 (CYP2E1), which is expressed primarily in the pericentral region of the liver, to its toxic metabolite N-acetyl-p-benzoquinoneimine (NAPQI). NAPQI irreversibly binds to the sulfhydryl group of reduced glutathione (GSH) as previously shown *in vitro* (Pierce et al., 2002[27]) and *in vivo* (Botta et al., 2006[5]). When GSH is depleted below critical thresholds, binding of NAPQI to protein targets and/or oxidative stress are responsible for cell death. Increased resistance to hepatotoxic effects (same dosage) caused by pretreatment leads to what is called 'autoprotection'. This response is accompanied in most cases with accumulation of extracellular matrix and compromised regeneration. The basic mechanisms of acute damage like e.g. inflammation, cell death and immunity (Jaeschke et al., 2012[19]) as well as contribution of neutrophils (Liu et al., 2006[23]) are clear. Several aspects, however, e.g. the contribution of oxidative and anti-oxidative processes, molecular and metabolic response dynamics along repeated APAP exposure still have to be elucidated. We here report that this organ has a specialized intrinsic hepatoprotective mechanism. This mechanism modulates the acute fulminant necroinflammatory process toward a fibrogenic wound healing response after the second exposure. Further investigations are required to identify the key players in this response shift.

## Materials and Methods

### Animals

The study was conducted on 8-10 week-old male Balb/c mice weighing 25-27 g. The experiments were carried out in accordance with the Guide for the Care and Use of Laboratory Animals published by the US National Institutes of Health (NIH Publication no. 85-23, revised in 1996) and approved by the local Ethical Committee. Mice were kept under a controlled temperature (22 ± 1 °C) and humidity (50 % ± 5 %), as well as under light/ dark cycles of 12 hours. Mice were acclimated for 4 days with free access to tap water and standard rodent chow. The mice had to fast overnight prior to acetaminophen (APAP) administration. 

### Experimental design

**Figure 1:**
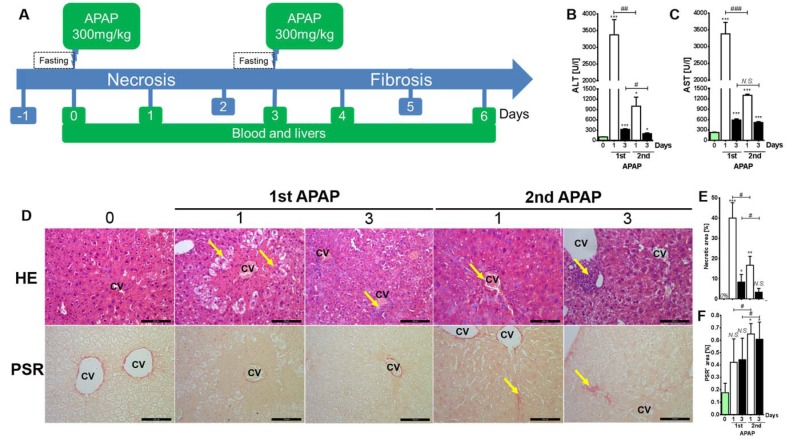
Liver response after the first and second APAP exposures. A) The experimental design is shown here. The mice were exposed to the first and the second APAP intoxication with a 3 day interval. Subsequently, blood and livers were collected at day 1 and at day 3. B and C) liver transaminases, namely ALT and AST, at day 1 and day 3. D) Liver tissue-based analysis by HE and PSR. Scale bars are 100 μm. E) and F) indicate the percentages of hepatocellular necrosis and fibrosis, respectively. ALT: Alanine aminotransferase; APAP: Paracetamol (Acetaminophen); AST: Aspartate aminotransferase; CV: central vein; HE: Hematoxylin&Eosin; PSR: Picrosirius red. Bars indicate means ± SD of 3-4 mice per time point. Statistical significance is indicated as follows: ***p<0.001, **p<0.01, and *p<0.05 compared to untreated mice; * replaced by # for comparison between the indicated bars.

Fifteen Balb/c mice (Male/8-10 weeks) were used in this study (Figure 1A(Fig. 1)). A control group (Ctrl) of three mice received just normal saline intraperitoneally (IP) and 12 mice received 300 mg/kg APAP (Sigma-Aldrich, #A5000). This dose was chosen based on a dose-dependent experiment on this mouse strain. The APAP was dissolved in normal saline and heated to 47 °C. The mice had to fast overnight prior to the injection of the first and second doses. 24 hours after the first APAP injection, the mice in the control group together with 3 mice (1 day after first dose) from the group that received injections were used to collect blood and liver. Two days later, 3 mice (3 days after the first dose) were sacrificed. The remaining mice received the second dose of APAP and one day later, three mice (1 day after second dose) were sacrificed. The last three mice were sacrificed on the sixth day (3 days after second dose). The blood as well as the livers were collected from all mice sacrificed. The blood was used to measure ALT and AST levels. The left liver lobe was fixed in para-formaldehyde (PFA) to be embedded in paraffin for an immunohistochemistry study. The right lobe was snap frozen at -80 °C for metabolomics studies using nuclear magnetic resonance spectroscopy (NMR) and molecular studies for gene expression profiling using real time PCR (Bio-Rad) as shown in Figure 1A(Fig. 1). 

### Liver function tests

The blood samples were collected from retro-orbital veins in heparinized tubes under ketamine anesthesia. Afterwards the heparinized blood was centrifuged at a speed of 3000x for 10 minutes to obtain plasma for ALT and AST (LABKIT-Barcelona) assays using an X monzaRandox reader.

### RNA isolation and transcriptomics

The isolation of RNA from the mouse liver tissue was performed using the RN-easy® plus Mini kit (Qiagen, MD, USA) according to the manufacturer´s protocol. Then, both RNA concentration and integrity were measured using a Nano UV-spectrophotometer (Quawell, CA, USA). Subsequently, the cDNA synthesis was performed using an RT2 First Strand Kit (Qiagen, MD, USA) according to the provider´s protocol. Then, a PAMM-120ZD-24 RT2profilerTM PCR array mouse fibrosis 96-well (Qiagen, MD, USA) was used as follows: The RT2 SYBR green was centrifuged for 10 seconds in the dark. The PCR master mix was prepared by mixing 1350 µl of 2x RT2 SYBR Green, 102 µl of cDNA and 1248 µl of RNase-free water in a 5 ml tube. Then, 25 µl of the PCR master mix was added to each well of a profilerTM PCR array, followed by centrifugation.

### Non-targeted metabolic profiling

High resolution magic angle spinning nuclear magnetic resonance spectroscopy (HRMAS-NMR) was used for non-targeted metabolic profiling. This technique rotates the sample at a “magic” angle with respect to the outer magnetic field in order to obtain NMR spectra, the resolution of which is comparable to that of liquid samples. The specimens were stored at -80 °C before preparation of samples for the NMR analysis. Measurements are done at a temperature of 4 °C to avoid decomposition of metabolites during the measurement. The samples are weighed in a dry (nitrogen atmosphere) and cold (-10 °C) environment, to avoid thawing and to exclude humidity. Liver tissue cores were acquired using a 1.3 mm punch (PFM Medical, Cologne, Germany). The tissue was weighed on a calibrated balance and then inserted carefully with uniform mass distribution into 33 µL disposable inserts (DI) (Bruker, Rheinstetten, Germany) for the 4 mm MAS rotor. Next, the DIs were filled with a solution of D_2_O and TSP at a concentration of 0.3% to obtain a lock signal and a reference for chemical shift calibration. The DI is closed with a cap and weighed again. A sucrose sample prepared from 10 μL of a H_2_O solution of 12.9 % concentration diluted with 1000 μL D_2_O is used for quantification using the ERETIC technique. The procedures are described in full by Gogiashvili et al. (2017[11]) and Hammad et al. (2018[16]). 

### Liver histopathology and immunostaining

The left liver lobe was fixed in para-formaldehyde and embedded in paraffin (Hammad et al., 2014[15]) for further histopathological investigations. To investigate necrotic areas and fibrotic indices, formalin-fixed paraffin embedded liver sections were stained by hematoxylin and eosin (HE) and PSR according to a standard protocol (Hammad et al., 2017[14]). The slides were scanned shortly after the staining procedure using the bright field microscope BX41. To assess the expression and localization of ECM producing cells, the metabolizing enzyme, the oxidative markers and the anti-oxidative enzymes, immunostaining was performed according to Hammad et al. (2014[15]) with alpha-smooth muscle actin (α-SMA, Abcam, 1:100), CYP2E1 (Sigma-Aldrich, HPA009128, 1:50), catalase (Santa Cruz, 1:50) and superoxide dismutase (Enzolifesciences, 1:100), respectively. A DAKO system was used for enzyme blocking and secondary antibodies according to the manufacturer's procedures. The activity of peroxidases was detected with diaminobenzidine (Sigma Aldrich, D5905). The slides were counterstained with hematoxylin. The immunoreactivity was visualized under a light microscope. Images of stained liver tissue were quantified employing ImageJ software (http://rsbweb.nih.gov/) to obtain the mean DAB signal intensity value. Five fields were chosen at random under 200-fold magnification. The percentages of positive signals were calculated from the total surface area of the images.

### Statistical analyses

Results are presented as mean ± S.D. When appropriate, a Student's t-test or a Mann-Whitney's test was used. The statistical significance is indicated as follows: ***p<0.0001, ***p<0.001, **p<0.01, and *p<0.05. * replaced by # for comparison between the indicated bars.

## Results

### A second APAP exposure induces liverfibrosis 

In contrast to a single exposure of hepatotoxic compounds like e.g. CCl_4_, repeated doses are always associated with liver fibrosis. In order to delineate the timing of fibrogenesis, mouse livers were administered once with APAP and a second exposure was applied 3 days later (Figure 1A(Fig. 1)). At only 24 h after the first dose, APAP induced white spots were recorded. Then standard necrosis indices and fibrosis scores were analyzed. Expectedly, the levels of liver transaminases, namely ALT and AST, were significantly increased at day 1 and at day 3 after the first APAP challenge (Figure 1B and C(Fig. 1); Supplementary Table 1) compared to untreated mice. Surprisingly, ALT and AST levels at day one after the second APAP exposure were significantly decreased compared to the corresponding time point of the first challenge. This finding indicates, that the acute fulminant liver injury is reduced after the second APAP exposure (Figure 1B and C(Fig. 1)). Instead of a pericentral necrosis (Figure 1D and E(Fig. 1); HE; yellow arrows; after first dose; Supplementary Table 1; Supplementary Figure 1) induced by a single APAP administration, inflammation and accumulation of irregular small shaped nuclei (Figure 1D and E(Fig. 1); HE; yellow arrows after second dose; Supplementary Table 1; Supplementary Figure 1) were observed after the second injection as indicated by HE staining. Furthermore, an extracellular matrix accumulation was reported at 1 and 3 days after the second APAP administration (Figure 1D and F(Fig. 1); PSR; yellow arrows; Supplementary Table 1; Supplementary Figure 1) as visualized by picrosirius red staining. The same results were obtained after chronic carbon tetrachloride (CCl_4_) intoxication in a different mouse strain (C57Bl6N) on blood and tissue levels. The given second APAP exposure shifts the liver response from an acute fulminant necroinflammatory process (induced by a single dose) to a fibrogenic wound healing response. 

### Alterations on genes, metabolites and protein levels after the second APAP administration

**Figure 2:**
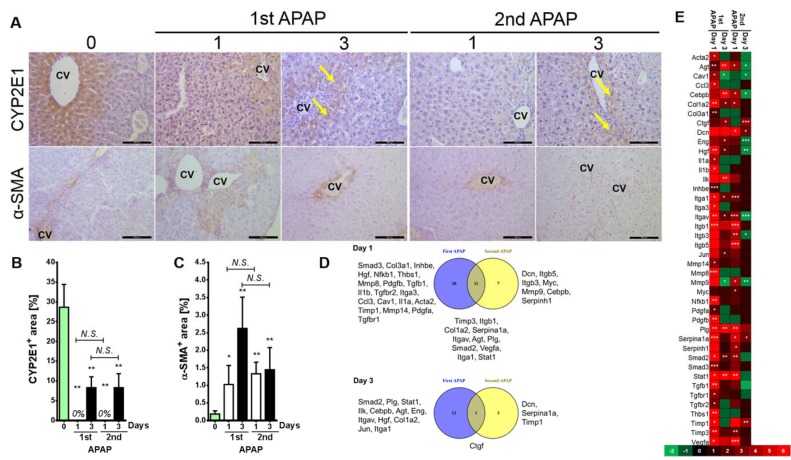
Tissue and molecular liver characterizations after the first and the second APAP intoxication. A) Using antibodies directed against CYP2E1 and α-SMA, IHC was performed. Scale bars are 100 μm. B) and C) indicate the percentages of CYP2E1 and α-SMA positive signals, respectively. Bars indicatemeans ± SD of 3-4 mice per time point. D) A Venn diagram shows significantly (p<0.05) upregulated genes in APAP exposed animals compared to untreated ones. E) A heat map for 42 significantly altered genes (at least in one time point compared with controls). ***p<0.001, **p<0.01, and *p<0.05 compared to untreated mice. APAP: Paracetamol (Acetaminophen); CV: central vein; CYP2E1: Cytochrome P450-2E1; α-SMA: alpha-smooth muscle actin.

**Figure 3:**
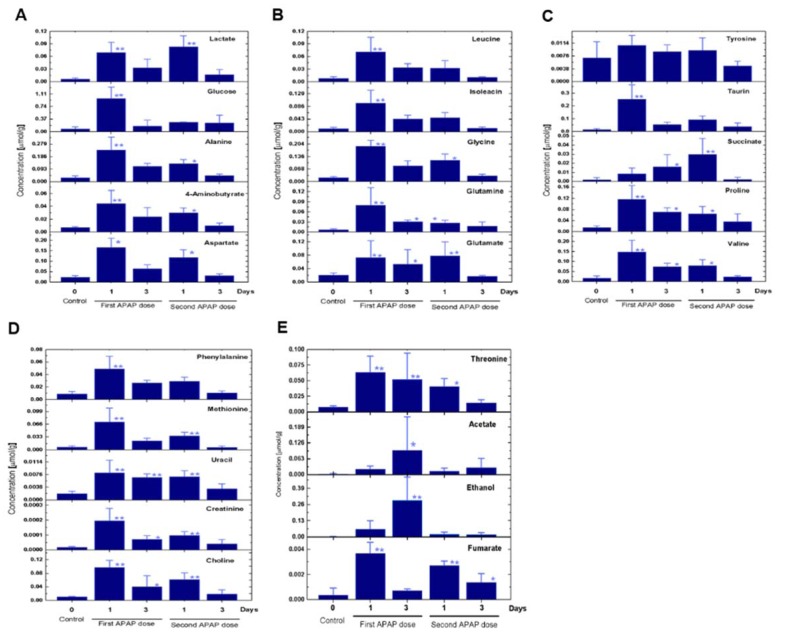
Non-targeted NMR-based metabolic profiling of APAP-exposed liver tissues. (A-E). 24 metabolites were identified in the liver tissue. The P-value indicates the statistical difference between the APAP treated livers and the control groups. Bars represent the means ± SD of 3 mice per group. Statistical significance is indicated as follows: **P<0.01, and *P<0.05 compared to untreated livers.

To gain a holistic insight into the alterations after the second APAP exposure, we assessed the expression patterns of metabolites, metabolic enzymes, ECM producing cells and fibrogenic genes. The cytochrome P450 2E1 (CYP2E1) expression on tissue level vanished after APAP administration (Figure 2A(Fig. 2)). This was expected, since a toxic dose of APAP kills the whole CYP2E1 positive fraction. Moreover, 3 days later CYP2E1 partially recovered around the necrotic zone (Figure 2A and B(Fig. 2), yellow arrows). Furthermore, an administration of APAP for a second time induced less necrosis (as indicated in Figure 1B-D(Fig. 1)), however, careful analysis of CYP2E1 positivity at day 3 after the second challenge revealed that the recovered CYP2E1^+^ cells are located directly around the central veins (Figure 2A(Fig. 2)). Furthermore, alpha-smooth muscle actin (α-SMA; the marker mostly used for ECM-producing cells) expression was higher at day 1 after the first APAP treatment compared to the same time point after the second dose (Figure 2A and C(Fig. 2)). Among 84 analyzed genes, 42 showed significant alterations in at least one time point compared to control mice (Figure 2D(Fig. 2)). Subsequent time points revealed that the liver is somehow adapted to the APAP toxicity. Obviously, alterations appeared at day 1 after the first APAP intoxication (Figure 2E(Fig. 2)). Among those significantly deregulated genes were stellate cell activation, ECM and TGFβ related ones as well as MMPs. Surprisingly, Mmp9, Itgb3, Hgf, Itgav, Cebpb, AGT and Eng were significantly downregulated compared to control mice at day 3 after the second APAP administration (Figure 2D(Fig. 2); Supplementary Table 2). The alterations between day 3 after the first and the second APAP exposure of Mmp8, Mmp9, Mmp14, Tgfβr1, Tgfβr2, Smad2 and Timp1 clearly show that a fibrosis had developed as a response to the second dose (Figure 2E(Fig. 2); Supplementary Table 2). A variety of non-significantly altered genes was also identified (Supplementary Figure 2; Supplementary Table 2). At day 3 after the second APAP exposure a particular expression pattern was found indicating that an adaptation process had been initiated. The metabolic profiling of liver tissue applying high resolution magic angle spinning nuclear magnetic resonance (HRMAS-NMR) revealed 24 metabolites (Figure 3(Fig. 3); Supplementary Table 3). Among those, alanine, glycine, isoleucine, methionine, uracil and valine were found to be significantly altered at day 3 after the second APAP dose compared to the corresponding first dose. Such results were reported after repeated CCl_4 _intoxication of black6 mice on gene and protein levels. Altogether, these results show that the second APAP administration is associated with different responses on the gene, the protein and the metabolite levels indicating that the liver has a remarkable memory for initially applied toxicants.

### The oxidative system is modulated during the second APAP injection

**Figure 4:**
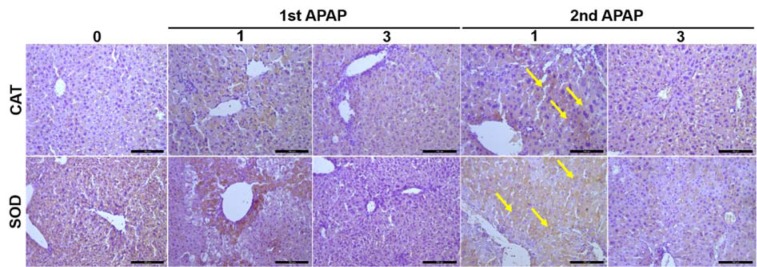
Tissue-based characterization of the oxidative system upon APAP intoxication. Using antibodies directed against CAT and SOD, we were able to visualize these targets in liver tissue. Scale bars are 100 µm. APAP: Paracetamol (Acetaminophen); CAT: Catalase; SOD: Sodium dismutase

To study the mechanisms that may be involved in the shift of the liver response after the second APAP challenge we investigated, whether the second APAP intoxication influences the oxidative system on the tissue level. Using antibodies directed against catalase, an antioxidant enzyme, revealed no signal in control livers, but most of the dead cells expressed catalase at day 1 after APAP intoxication (Figure 4(Fig. 4)). Careful analysis indicates that several catalase positive hepatocytes were observed at day 1 and to some extent at day 3 after the second APAP exposure (Figure 4(Fig. 4), yellow arrows). Similar to catalase, high levels of superoxide dismutase, a potent antioxidant enzyme, were expressed at day 1 and at day 3 after the second APAP exposure (Figure 4(Fig. 4), yellow arrows; Supplementary Figure 1). In conclusion, the oxidative system is modified during the second APAP administration toward a hepatoprotective process. 

## Discussion

Clinically, APAP is a widely used drug for the treatment of pain and fever (summarized in a recent review (Lee, 2017[22])). Furthermore, APAP is a well-studied model for acute liver intoxication and regeneration (Alempijevic et al., 2017[1]; Gong et al., 2018[12]). Little is known, however, about the fibrogenicity of APAP. The administration of repeated doses of APAP has been recently reported to induce liver fibrosis (Bai et al., 2017[3]; Eakins et al., 2015[9]; Huang et al., 2018[17]; Yan et al., 2018[34]). In the current study, different liver responses to the first and the second APAP intoxication were observed. A significant reduction of liver enzymes, namely ALT and AST, as well as the formation of hepatocellular necrotic areas were reported after the second APAP exposure, as was previously shown (Buttar et al., 1976[6]; Poulsen and Thomsen, 1988[28]). These findings confirm the adaptive liver responses and hepatoprotective hypothesis suggested by Dalhoff and colleagues (2001[8]) and Eakins and co-workers (2015[9]). These autoprotective responses against the second APAP exposure were previously reported (Rudraiah and Manautou, 2016[31]; Rudraiah et al., 2014[32]) and observed after carbon tetrachloride long-term intoxication which suggests that this is a generalized liver response. Surprisingly, in this study, as another toxic response, fibrogenesis is observed as indicated by PSR and alpha-SMA staining. 

We hypothesize that this switching from necroinflammatory fulminating intoxication to a fibrogenesis wound healing response represents a generalized feature of liver adaptation for maintaining critical hepatic functions. In this study, a multi-level analysis including organ transcriptomics, metabolomics as well as tissue scale investigations reveals the alteration between the first and the second APAP exposures. On the transcriptomics level, the significant changes of gene expression of Mmp9, Tgfβ, observed after the first dose, indicate the response shift to liver fibrosis. Owing to the hepatic adaptation mechanisms, however, several genes recovered to the basal levels and only few genes were upregulated after the second dose like Dcn and Timp1. Overexpression of Dcn has been studied as a physiological inhibitor of Tgfβ that promotes tissue regeneration and decreases fibrosis (Thu et al., 2016[33]). Timp1 overexpression could be responsible for the downregulation of Mmps as they were found to be inhibited in long-term APAP administration studies (Bai et al., 2017[3]). These findings are well correlated with the identified pharmacological and toxicological end point signatures for rats that were repeatedly exposed to APAP (Natsoulis et al., 2008[24]). In addition, significant reduction of the concentration of some metabolites excreted to the microenvironment of liver cells seen 3 days after administration of the second dose could also indicate a hepatoadaptation to APAP that decreases the release of metabolites to their microenvironment. In accordance with these findings, APAP induces a similar induction pattern of metabolites in Zebra fish larvae (Huang et al., 2017[18]). While there are no metabolic profiling data available after repeated APAP exposures, Kyriakides and co-workers, however, reported some metabolic alterations of e.g. valine, uracil, and aspartate at the early time points 3 and/or 6 hours after APAP administration (Kyriakides et al., 2016[20]). The transcriptomics and metabolomics approaches reveal the behavior of the liver upon the first and the second APAP exposure. This multiOMICs approach shows how the system responds to intoxication/regeneration/ fibrosis incidents.

The role of phase II toxicokinetics in APAP detoxification has been well studied. Particularly, glutathione and NAPQI (a toxic APAP metabolite) binding has been described as an efficient detoxifying mechanism. Therefore, we investigated the availability of antioxidant enzymes like e.g. SOD and catalase in mice exposed to APAP. Surprisingly, we observed an increased availability of the aforementioned enzymes near injured areas after the second APAP exposure. It was previously shown that SOD2 depletion leads to mitochondrial dysfunction, DNA sequestration and in turn increases the APAP-induced hepatotoxicity (Ramachandran et al., 2011[30]). Thus, upregulation of an anti-oxidative system like e.g. SOD2 by a second APAP administration might explain this protective phenotype.

## Conclusion

There are changes in transcriptomics, metabolomics and tissue characteristics indicating a significant difference in the liver response between the first and second APAP intoxication. This response is a necroinflammatory fulminant liver injury in case of the first dose and a fibrogenesis via accumulation of extracellular matrix after the second APAP dose. The most possible scenarios are that i) activated hepatic stellate cells (HSCs), i.e. ECM producing cells, seem to be biphenotypical cells. After the first APAP intoxication HSCs become active and secrete ECM, however, after the second APAP administration there is an oversecretion of ECM, or ii) APAP-induced hepatotoxicity is CYP2E1-dependent. It is well accepted that CYP2E1 is downregulated after the first challenge and recovers in 2-4 weeks. This may explain both the less fulminant liver damage and the increase in ECM deposition. Further studies are required to mechanistically understand this bipotential liver response.

## Notes

Mohammad AlWahsh and Seddik Hammad (Molecular Hepatology Section, Department of Medicine II, Medical Faculty Mannheim, Heidelberg University, Theodor-Kutzer-Ufer 1-3, 68167 Mannheim, Germany; Tel: +49 621 383 5603, E-mail: seddik.hammad@medma.uni-heidelberg.de) contributed equally as corresponding authors.

## Acknowledgement

The authors thank Kerry Cherise Gould (UMM-University of Heidelberg) for her technical assistance in IHC staining and microscopy.

## Author contributions

MA, AO, LH, AT, JL, SH, AA and FM conceived the study, SH, MA and LH performed data analyses and wrote the manuscript. JL and SH performed a critical revision of the manuscript. RH, TA, SH and SD provided supervisory support and corrected the manuscript. All authors read the final version of the paper.

## Financial support

SH and SD were supported by the BMBF (German Federal Ministry of Education and Research) Project LiSyM (Grants PTJ-FKZ: 031 L0043) and e:Bio - Modul-II : MS_DILI. MA, AO, AT, JL and RH were supported by the Ministerium für Innovation, Wissenschaft und Forschung des Landes Nordrhein-Westfalen, the Senatsverwaltung für Wirtschaft, Technologie und Forschung des Landes Berlin, and the Bundesministerium für Bildung und Forschung. FM receives support from Alexander von Humboldt-Stiftung. This work is also supported by Al-Zaytoonah University of Jordan.

## Conflict of interest

The authors declare that there is no conflict of interest regarding the publication of this paper.

## Data availability statement

The data used to support the findings of this study can be found in the supplementary material.

## Supplementary Material

Supplementary material
