# Supplementary material for: Second exposure to acetaminophen overdose is associated with liver fibrosis in mice
Source: EXCLI J. 2019 Feb 6;18:51–62. (PMC6449668)

EXCLI Journal 2019;18:51-62 – ISSN 1611-2156
Received: November 21, 2018, accepted: January 28, 2018, published: February 06, 2019

Original article:

SECOND EXPOSURE TO ACETAMINOPHEN OVERDOSE IS ASSOCIATED WITH LIVER FIBROSIS IN MICE

Mohammad AlWahsh[1,2*], Amnah Othman[2], Lama Hamadneh[1], Ahmad Telfah[2], Jörg Lambert[2], Suhair Hikmat[1], Amin Alassi[1], Fatma El Zahraa Mohamed[3,4], Roland Hergenröder[2], Tariq Al-Qirim[1], Steven Dooley[3], Seddik Hammad[3,5*]

[1] Department of Pharmacy, Faculty of Pharmacy, Al-Zaytoonah University of Jordan, P.O. Box 130, Amman 11733, Jordan
[2] Leibniz-Institut für Analytische Wissenschaften - ISAS - e.V., Bunsen-Kirchhoff-Straße 11, 44139 Dortmund, Germany
[3] Molecular Hepatology Section, Department of Medicine II, Medical Faculty Mannheim, Heidelberg University, 68167-Mannheim, Germany
[4] Department of Pathology, Faculty of Medicine, Minia University, 11432-Minia, Egypt
[5] Department of Forensic Medicine and Veterinary Toxicology, Faculty of Veterinary Medicine, South Valley University, 83523-Qena, Egypt

* Corresponding authors: Mohammad AlWahsh: Leibniz Institut für Analytische Wissenschaften - ISAS e.V., Bunsen-Kirchhoff-Straße 11, 44139 Dortmund, Germany, Tel: +49 231 1392 192. E-mail: mohammad.al-wahsh@isas.de
Seddik Hammad: Molecular Hepatology Section, Department of Medicine II, Medical Faculty Mannheim, Heidelberg University, Theodor-Kutzer-Ufer 1-3, 68167 Mannheim, Germany, Tel: +49 621 383 5603. E-mail: seddik.hammad@medma.uni-heidelberg.de

http://dx.doi.org/10.17179/excli2018-1920

This is an Open Access article distributed under the terms of the Creative Commons Attribution License (http://creativecommons.org/licenses/by/4.0/).

Supplementary Table 1: Liver enzymes level, necrosis index and fibrosis score per mouse. The average data are presented in Figure 1. ALT: Alanine aminotransferase; APAP: Acetaminophen; AST: Aspartate aminotransferase; PSR: Picrosirius red.

			**1st APAP**		**2nd APAP**	
	Mouse ID	0d	1d	3d	1d	3d
**ALT (U/I)**	#1	102	2500	300	1472	160
	#2	105	3596	360	960	200
	#3	112	4030	320	560	240
**AST (U/I)**	#1	217	2700	644	1250	480
	#2	220	3660	588	1300	520
	#3	250	3788	532	1360	560
**Necrotic area (%)**	#1	0	25	8	15	1
	#2	0	50	2	10	7
	#3	0	45	15	25	2
**PSR$^+$ area (%)**	#1	0.125	0.055	0.632	0.698	0.635
	#2	0.325	0.686	0.096	0.766	0.833
	#3	0.076	0.524	0.600	0.486	0.357

Supplementary Table 2: Deregulated genes in the liver upon APAP administration. Numbers are averages of 3 mice compared with untreated mice. (-) refers to downregulated genes. These data are presented in figure 2D, 2E and supplementary Figure 2.

	**Fold Change (Vs untreated mice)**			
	**1st APAP**		**2nd APAP**	
	**1 day**	**3 days**	**1 day**	**3 days**
Acta2	3.694	-1.196	1.421	-1.551
Actb	-1.079	-1.883	-1.611	-1.412
Agt	1.866	4.797	2.872	-1.519
Akt1	1.847	1.582	1.199	-1.883
B2m	-1.223	1.471	-1.041	-1.208
Bcl2	1.157	-1.188	-1.213	1.062
Bmp7	1.157	-1.188	-1.213	1.062
Cav1	3.238	-1.997	-1.171	-1.583
Ccl11	1.157	-1.188	-1.213	1.062
Ccl12	1.157	-1.188	-1.213	1.062
Ccl3	4.724	-1.188	-1.213	1.062
Ccr2	1.157	-1.188	-1.213	1.062
Cebpb	2.878	7.023	3.187	-1.583
Col1a2	14.877	2.177	2.774	1.037
Col3a1	1.490	-1.188	-1.213	1.062
Ctgf	2.085	2.570	-1.147	2.765
Cxcr4	1.157	-1.188	-1.213	1.062
Dcn	10.630	7.195	8.039	2.341
Edn1	1.157	-1.188	-1.213	1.062
Egf	1.157	-1.188	-1.213	1.062
Eng	2.540	2.253	1.793	-1.332
Fasl	1.157	-1.188	-1.213	1.062
Gapdh	1.521	-1.131	1.272	1.756
Grem1	1.157	-1.188	-1.213	1.062
Gusb	-1.235	1.096	-1.093	1.062
Hgf	9.514	1.996	1.212	-1.662
Hsp90ab1	1.072	1.321	1.441	-1.093
Ifng	1.157	-1.188	-1.213	1.062
Il10	1.157	-1.188	-1.213	1.062
Il13	1.157	-1.188	-1.213	1.062
Il13ra2	1.157	-1.188	-1.213	1.062
Il1a	3.732	-1.188	-1.213	1.062
Il1b	9.448	-1.188	-1.070	1.062
Il4	1.157	-1.188	-1.213	1.062
Il5	1.157	-1.188	-1.213	1.062
Ilk	7.621	6.373	2.474	1.062
Inhbe	1.505	-1.188	1.037	1.062
Itga1	6.612	2.398	2.261	1.062
Itga2	1.157	-1.188	-1.213	1.062
Itga3	5.502	-1.188	1.170	1.062
Itgav	8.084	1.982	2.745	-1.745
Itgb1	22.085	3.842	6.878	-1.027
Itgb3	15.455	2.277	3.220	-1.260
Itgb5	10.091	2.717	4.444	-1.016
Itgb6	1.157	-1.188	-1.213	1.062
Itgb8	1.157	-1.188	-1.213	1.062
Jun	3.317	1.720	2.017	1.062
Lox	1.157	-1.188	-1.213	1.062

	**Fold Change (Vs untreated mice)**			
	**1st APAP**		**2nd APAP**	
	**1 day**	**3 days**	**1 day**	**3 days**
Ltbp1	1.157	-1.188	-1.213	1.062
MGDC	1.157	-1.188	-1.213	1.062
Mmp13	1.157	-1.188	-1.213	1.062
Mmp14	2.321	1.241	1.661	1.062
Mmp1a	1.157	-1.188	-1.204	1.062
Mmp2	1.157	-1.188	-1.213	1.062
Mmp3	1.157	-1.188	-1.213	1.062
Mmp8	7.945	-1.188	-1.213	1.062
Mmp9	19.054	-1.909	3.110	-1.514
Myc	1.591	1.183	1.599	1.062
Nfkb1	12.042	1.268	1.582	1.062
Pdgfa	1.945	-1.188	1.022	1.062
Pdgfb	7.160	-1.188	-1.213	1.062
Plat	1.157	-1.188	-1.213	1.062
Plau	1.189	-1.188	-1.213	1.062
Plg	26.630	44.231	14.795	3.427
PPC	1.694	-2.011	-1.751	1.195
PPC	1.210	-1.403	-1.336	1.051
PPC	1.223	-1.656	-1.139	1.138
RTC	2.297	-4.341	-1.600	-2.776
RTC	7.781	-1.739	1.387	-1.709
RTC	3.931	-2.392	-1.056	-1.369
Serpina1a	15.617	2.374	3.803	2.492
Serpine1	1.429	-1.188	-1.213	1.062
Serpinh1	7.362	-1.048	2.643	1.062
Smad2	9.481	1.895	2.726	-1.175
Smad3	2.621	1.030	2.192	1.062
Smad4	1.485	1.216	1.191	1.062
Smad6	1.414	1.294	-1.213	1.062
Smad7	1.548	1.150	-1.213	1.062
Snai1	1.157	-1.188	-1.213	1.062
Sp1	1.157	-1.188	-1.213	1.062
Stat1	8.907	7.527	17.172	-1.063
Stat6	1.157	-1.188	-1.213	1.062
Tgfb1	10.056	1.187	1.690	-2.511
Tgfb2	1.157	-1.188	-1.213	1.062
Tgfb3	1.157	-1.188	-1.213	1.062
Tgfbr1	3.193	1.456	1.077	-1.059
Tgfbr2	2.497	-1.188	1.869	1.062
Tgif1	1.157	-1.188	-1.213	1.062
Thbs1	8.515	-1.188	-1.213	1.062
Thbs2	1.157	-1.188	-1.213	1.062
Timp1	4.258	-1.188	4.698	3.220
Timp2	2.928	1.066	-1.213	1.062
Timp3	4.347	2.124	2.147	1.062
Timp4	1.157	-1.188	-1.213	1.062
Tnf	1.157	-1.188	-1.213	1.062
Vegfa	17.753	5.053	6.974	1.431

Supplementary Table 3: Metabolic profiling in the liver upon APAP administration. Metabolites level in the liver per mouse is presented. The average data are presented in Figure 3.

Metabolite (µMol/g tissue)	Untreated			1st APAP						2nd APAP					
				1 day			3 days			1 day			3 days		
	#1	#2	#3	#1	#2	#3	#1	#2	#3	#1	#2	#3	#1	#2	#3
**4-Aminobutyrate**	0.0077	0.0061	0.0086	0.0464	0.0640	0.0236	0.0238	0.0098	0.0381	0.0305	0.0203	0.0397	0.0101	0.0142	0.0060
**Acetate**	0.0022	0.0016	0.0034	0.0089	0.0244	0.0317	0.0351	0.0071	0.2483	0.0123	0.0121	0.0152	0.0075	0.0048	0.0693
**Alanine**	0.0247	0.0218	0.0461	0.1285	0.2936	0.2858	0.1307	0.0906	0.1222	0.0847	0.1483	0.1682	0.0538	0.0508	0.0349
**Aspartate**	0.0226	0.0143	0.0313	0.1727	0.2078	0.1216	0.0633	0.0468	0.0837	0.1110	0.0718	0.1710	0.0394	0.0320	0.0222
**Choline**	0.0103	0.0093	0.0121	0.0739	0.1095	0.1095	0.0785	0.0229	0.0198	0.0459	0.0553	0.0850	0.0292	0.0221	0.0054
**Creatinine**	0.0000	0.0000	0.0000	0.0001	0.0003	0.0002	0.0001	0.0000	0.0001	0.0001	0.0001	0.0001	0.0000	0.0001	0.0000
**Ethanol**	0.0019	0.0016	0.0023	0.1383	0.0353	0.0107	0.2801	0.4845	0.1125	0.0248	0.0353	0.0041	0.0333	0.0057	0.0197
**Fumarate**	0.0005	0.0002	0.0004	0.0020	0.0056	0.0027	0.0007	0.0008	0.0007	0.0026	0.0023	0.0032	0.0024	0.0014	0.0004
**Glucose**	0.0259	0.1493	0.0804	0.6531	1.3097	0.9844	0.0402	0.1083	0.3693	0.2662	0.2819	0.2990	0.2935	0.4703	0.0215
**Glutamate**	0.0209	0.0143	0.0272	0.0739	0.0218	0.1230	0.1035	0.0350	0.0220	0.0301	0.0782	0.1265	0.0171	0.0150	0.0206
**Glutamine**	0.0080	0.0055	0.0093	0.0245	0.1271	0.0887	0.0337	0.0262	0.0346	0.0178	0.0301	0.0354	0.0133	0.0321	0.0079
**Glycine**	0.0213	0.0149	0.0284	0.1666	0.2287	0.1829	0.1079	0.0557	0.0944	0.0611	0.1460	0.1415	0.0363	0.0391	0.0214
**Isoleucine**	0.0102	0.0065	0.0157	0.0469	0.1190	0.1261	0.0584	0.0383	0.0345	0.0251	0.0536	0.0658	0.0161	0.0118	0.0101
**Lactate**	0.0068	0.0042	0.0089	0.0732	0.0919	0.0430	0.0443	0.0111	0.0454	0.1018	0.1065	0.0429	0.0307	0.0114	0.0088
**Leucine**	0.0092	0.0047	0.0116	0.0309	0.0880	0.0941	0.0440	0.0281	0.0303	0.0122	0.0457	0.0434	0.0126	0.0100	0.0091
**Methionine**	0.0059	0.0048	0.0114	0.0323	0.0812	0.1029	0.0159	0.0288	0.0241	0.0346	0.0350	0.0371	0.0064	0.0102	0.0042
**Phenylalanine**	0.0080	0.0056	0.0134	0.0269	0.0543	0.0660	0.0230	0.0314	0.0264	0.0200	0.0311	0.0368	0.0124	0.0124	0.0080
**Proline**	0.0135	0.0117	0.0215	0.0616	0.1457	0.1444	0.0852	0.0546	0.0732	0.0256	0.0781	0.0924	0.0233	0.0690	0.0169
**Succinate**	0.0047	0.0003	0.0008	0.0055	0.0045	0.0156	0.0135	0.0046	0.0308	0.0111	0.0436	0.0346	0.0022	0.0000	0.0046
**Taurine**	0.0174	0.0119	0.0227	0.1334	0.2771	0.3526	0.0468	0.0446	0.0755	0.0500	0.1137	0.1150	0.0291	0.0699	0.0172
**Threonine**	0.0088	0.0047	0.0087	0.0369	0.0884	0.0651	0.1003	0.0299	0.0260	0.0186	0.0502	0.0500	0.0120	0.0117	0.0199
**Trimethylamine**	0.0101	0.0000	0.0001	0.0006	0.0014	0.0015	0.0000	0.0258	0.0293	0.0010	0.0000	0.0004	0.0000	0.0000	0.0099
**Tyrosine**	0.0125	0.0032	0.0056	0.0090	0.0091	0.0142	0.0084	0.0112	0.0072	0.0037	0.0112	0.0131	0.0058	0.0052	0.0033
**Uracil**	0.0023	0.0011	0.0023	0.0061	0.0058	0.0125	0.0075	0.0072	0.0055	0.0042	0.0087	0.0079	0.0034	0.0048	0.0020
**Valine**	0.0139	0.0115	0.0295	0.0816	0.1682	0.1938	0.0936	0.0612	0.0680	0.0302	0.1012	0.1077	0.0275	0.0264	0.0184

Supplementary Figure 1: Formalin-fixed livers were processed and stained with HE, PSR and SOD to visualize and quantify hepatocellular necrosis, Extracellular matrix deposition and anti-oxidant system. Scale bars are 200µm for HE and 100µm for PSR and SOD.

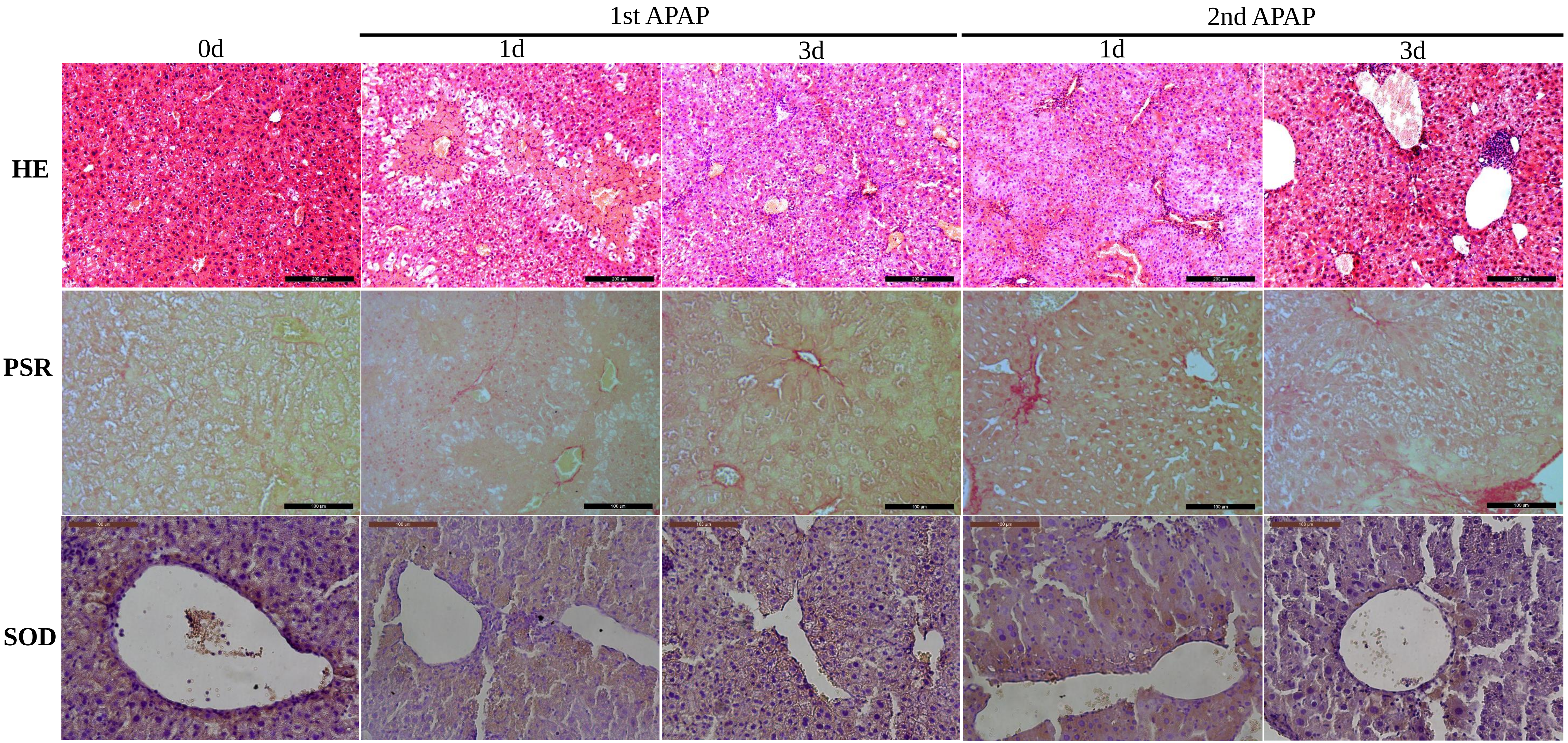

Supplementary Figure 2: A heat map for unaltered genes upon APAP intoxication.

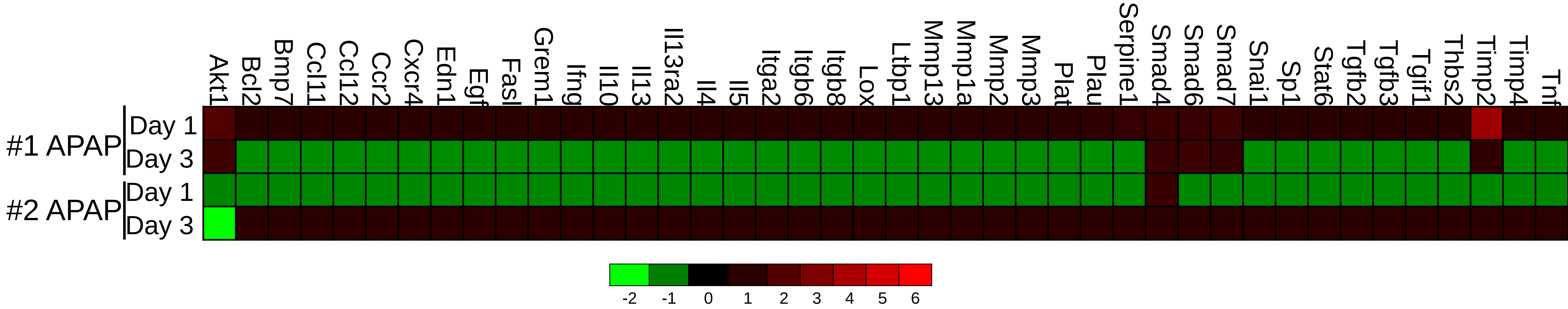

Supplement: Supplementary material [file EXCLI-18-51-s-001.pdf]
